# Polymorphisms in Oxytocin and Vasopressin Receptor Genes as a Factor Shaping the Clinical Picture and the Risk of ASD in Males

**DOI:** 10.3390/brainsci13040689

**Published:** 2023-04-20

**Authors:** Krzysztof M. Wilczyński, Aleksandra Stasik, Lena Cichoń, Aleksandra Auguściak-Duma, Małgorzata Janas-Kozik

**Affiliations:** 1 Department of Developmental Age Psychiatry and Psychotherapy SUM, 40-055 Katowice, Poland; 2 John Paul II Children’s and Family Health Center, sp. z o.o, 41-218 Sosnowiec, Poland; 3 Department of Molecular Biology and Genetics SUM, 40-752 Katowice, Poland

**Keywords:** autism, oxytocin, vasopressin

## Abstract

Autism spectrum disorders (ASD) are a heterogeneous group of disorders affecting virtually every population, regardless of their ethnic or socioeconomic background. Their pathogenesis is multifactorial, based on interactions between genetic and environmental factors. The key symptom of ASD are deficits in social communication, which are the basis of many difficulties in everyday functioning. The aim of the presented study was to analyze the clinical picture of social cognition deficits in boys with autism spectrum disorders and to relate its elements with the frequency of alleles of selected polymorphisms within the oxytocin receptor (OXTR) and vasopressin receptor 1A (AVPR1A) genes. The study included 58 boys with IQ > 90, who were divided into two groups based on a confirmed or excluded ASD diagnosis based on the DSM-5 and ICD-10 criteria and then using the ADOS-2 protocol. The results indicated that polymorphism rs10877969 (T) within the AVPR1a gene was the only one to show a statistically significant association with a higher risk of autism spectrum disorders and has an impact on clinical presentation in the ADOS-2 study, primarily in terms of the social affect subscale. Polymorphisms in the OXTR gene showed no significant association with ASD risk and severity of autistic traits in the ADOS-2 study. In the group of people with ASD and those who are neurotypical, the rs53572 (A) genotype in the OXTR gene significantly increased the severity of the clinical picture of social cognition disorders in reading mind in the eyes test (RMiE) and empathy quotient (EQ) studies.

## 1. Introduction

The ability to establish and maintain relationships, which is a sophisticated result of human evolution, is one of the key factors influencing mental health and quality of life. Difficulties in social cognition observed in autism spectrum disorder, including deficits in theory of mind and empathy, as well as unskillful reading of non-verbal communication (e.g., through facial expressions) are among the elements responsible for the characteristic symptoms of ASD, such as difficulties in understanding metaphors, sharing interests, and maintaining mutual interaction with another person [1,2]. Therefore, the question of the proper nature of communication disorders observed in the course of ASD, as well as their possible neurochemical basis remains of interest. In this respect, for several years, researchers have been focusing on two neurohormones that have a comprehensive effect on human functioning—oxytocin (OXT) and vasopressin (AVP). Among all the species in which the presence of these nonapeptides is observed, regardless of the class, they have three characteristics [3]:

1. They are secreted within the nervous system;
2. Their function is modulated by sex hormones, and due to that, sexual dimorphism in their function is observed;
3. They are crucial for social/reproductive behavior.

This, combined with the significant evolutionary conservatism of the structure of these nonapeptides, supports the hypothesis that they may play a key role in the social functioning of humans and other species [4]. However, studies of oxytocin levels in neurotypical and neuroatypical people show conflicting results [5]. This, in turn, drew researchers’ attention to the structure and expression of oxytocin receptors (oxytocin receptor (OXTR)) and arginine vasopressin 1A and 1B (arginine vasopressin receptor 1A/1B (AVPR1A/1B)). In a 2015 meta-analysis, LoParo and Waldman analyzed seven studies on single-nucleotide polymorphism (SNPs) in the OXTR gene comprising a total of 16 different SNPs. As possible risk factors for ASD, they proposed polymorphisms: rs7632287 (A allele), rs237887 (A allele), rs2268491 (T allele), and rs2254298 (A allele) [6]. In the case of the AVPR gene, there are only a few publications in the literature that seem to indicate a relationship between the social cognition disorders observed in the course of ASD and polymorphisms in the AVPR1a gene [7,8,9]. Of particular interest here are the polymorphisms rs7294536 (allele A) and rs10877969 (allele A), the possible relationship of which was shown in a recent study from 2017 by Yang et al. [10]. Unfortunately, due to the significant heterogeneity of the results, the current literature does not allow drawing unambiguous conclusions, not only in the context of their existence, but also as to the alleles that would be associated with social cognition disorders in ASD. In addition, there is a lack of data in the literature that would simultaneously analyze polymorphisms in genes related to the oxytocinergic and vasopresynergic systems in the same group of patients. 

The aim of the present study was to analyze the relationship between selected polymorphisms within the OXTR i and AVPR1A genes with the risk and severity of the clinical picture of ASD symptoms in a group of boys with this neurodevelopmental disorder and those who are neurotypical. 

## 2. Materials and Methods

### 2.1. Study Participants

The research was conducted at the Department of Psychiatry and Psychotherapy of Developmental Age of the John Paul II Children’s and Family Health Center in Sosnowiec Sp. z o.o. in cooperation with the Department of Molecular Biology of the Medical University of Silesia in Katowice. Participants for the study group were recruited from among the patients of the John Paul II Children’s and Family Health Center in Sosnowiec outpatient and inpatient clinics of the Department of Psychiatry and Psychotherapy of Developmental Age. Participants for the control group were recruited from among students of schools in the Silesian Voivodeship. The study population consisted of a monoethnic Polish group of children. Informed consent to participate in the study was obtained after information had been provided from both the parents and the participants themselves.

The criteria for inclusion in the study group included ASD diagnosis confirmation by the ADOS-2 protocol. Exclusion criteria for the study and control groups included: (1) concomitant diagnosis of another psychiatric disorder, (2) age under 12 and over 19 years, (3) concomitant intellectual disability, (4) epilepsy, (5) known genetic, neurometabolic, etc., background of observed symptoms (e.g., fragile X syndrome), (6) poor somatic status (significant somatic symptoms affecting the ability to participate in the study—e.g., fever in the course of infection), (7) diagnosis of serious liver, kidney, or heart disease, and (8) hypothyroidism. In addition, in the case of the control group, the exclusion criteria were: (1) suspicion or diagnosis of ASD in the history and (2) the presence of a person with a diagnosis of ASD among 1st- and 2nd-degree relatives. 

The data collected in the study were pseudonymized. The study included 58 boys with IQ > 90 who were divided into two groups based on a confirmed or excluded ASD diagnosis based on the DSM-5 and ICD-10 criteria, then using the ADOS-2 protocol. The study group included 37 boys diagnosed with ASD. The mean age in this group was 14.56 years (95%CI: ±13.88–15.25 years). The control group included 21 boys who, in the mental status study, did not meet the criteria for a diagnosis of ASD and did not meet the cut-off threshold for ASD in the ADOS-2 study. The mean age in this group was 17.04 years (95%CI: 15.85–18.23 years). The mean age difference between the control and study groups was statistically significant with *p* < 0.05 (Mann–Whitney U test). 

### 2.2. Psychometric Analysis 

All participants were tested with the ADOS-2 protocol using modules corresponding to the age and language level of the participants. The analyses focused primarily on the comparative score (ADOS:WP), the domain of social affect (ADOS:SA), and the domain of repetitive and stereotyped patterns of behavior and interests (ADOS:RRB). In addition, the study used:

- The “Reading mind in the eyes” test: This is a tool designed by Baron–Cohen, which in the version applied in the present study consists of 28 images presenting eye slices surrounded by 4 definitions of mental state, from which the subject has to choose one, corresponding to the presented figure. The test is performed without a time limit [11,12].
- The “Empathy Quotient” test is a tool designed by Baron–Cohen (translated by Jankowiak-Siuda et al. from 2017), allowing determining individual differences in the ability to empathize. The presented work uses the version of Wakabayashi et al. containing 22 test items [13].

### 2.3. Consent of the Bioethics Committee and Source of Funding

The study was conducted with the consent of the Bioethics Committee of SUM issued by Resolution No. CDF/0022/KB1/123/18/19 of 08.01.2019. The costs of the research were covered by the Medical University of Silesia under statutory employment contracts, Nos. KNW-2-K18/D/9/N and KNW-1-178/N/9/K. 

### 2.4. Molecular Analysis 

Two selected polymorphisms of the OXTR gene and two selected polymorphisms of the AVPR1a gene were analyzed. In the case of the oxytocin receptor, the following polymorphisms were included: rs53576 (G/A) and rs2254298 (A/G); for the arginine vasopressin receptor subtype 1A: rs7294536 (T/C) and rs10877969 (T/C).

Analysis of DNA polymorphisms: DNA was isolated from 200 μL of peripheral blood collected on EDTA (here, the name of blood collection tubes) using the GeneMatrix Quick Blood DNA Puriffication Kit (EuRX, Gdańsk, Poland) in accordance with the manufacturer’s guidelines. Qualitative and quantitative evaluation of DNA was performed spectrophotometrically on the NanoDrop 2000 device (Thermo Fisher Scientific, Waltham, MA, USA). Genotyping analysis was then performed in two repetitions on the LightCycler480 II thermal cycler (Roche, Mannhaim, Germany) using TaqMan SNP Genotyping Assay probes (for rs3796863: c_1216944_10; for rs6449197: c_3109652520) (Applied Biosystems, Waltham, MA, USA) and TaqPath ProAmp Master Mix (Thermo Fisher Scientific, USA) in accordance with the manufacturer’s guidelines.

### 2.5. Statistical Analysis 

The statistical analysis was performed using Statistica Version 13 software (StatSoft Polska Sp. z o.o., Krakow, Poland). The assumed level of statistical significance was a = 0.05. The analysis of the allele frequency of selected polymorphisms was carried out using the frequency formula resulting from the Hardy–Weinberg law determining the relationship between allele frequency and genotype frequency in the population. 

Formula used:

(p + q)^2^ = p^2^ + 2pq + q^2^ assuming p + q = 1

In this study, “p” corresponds to the frequency of the wild (major) allele and “q” to the frequency of the mutant (minor) allele. Intergroup comparisons for quantitative variables were made using the Mann–Whitney U test and for qualitative variables using the χ^2^ test. The assumed significance level was α = 0.05.

## 3. Results

The mean total ADOS-2 score in the study group was 16.42 points (95%CI: 15.01–17.82 points), which translated into an average comparative score of 7.92 points (95%CI: 7.38–8.45 points; min/max: 5/10 points; *p* < 0.05). In the control group, these scores were 3.19 points (95%CI: 2.38–3.99 points) and 1.38 points (95%CI: 0.89–1.86 points; *p* < 0.05). In the social affect subscale, the average score was 13.86 points (95%CI: 12.69–15.04) and 2.76 points (95%CI: 1.92–3.60; *p* < 0.05), respectively, and for repetitive patterns of behavior, 2.57 points (95%CI: 2.03–3.12 points) and 0.42 points (95%CI: 0.12–0.73 points; *p* < 0.05). 

The distribution and frequency of alleles of selected polymorphisms in the study and control groups is presented in Table 1. In the comparative analysis of structure indicators in the allele range of the studied polymorphisms between the control and study groups, a statistically significant difference in frequency was observed only in the case of SNP rs10877969 (χ2 = 12.77 with *p* = 0.0004; x_C_(tested) = 0.34 vs. x_C_(control) = 0.64). In the remaining polymorphisms, no significant differences in the distribution of the studied alleles were observed. Similarly, in the logistic regression analysis, the only significant predictor of belonging to the study group (confirmed diagnosis of ASD) was the polymorphism rs10877969 (AVPR1a; minor allele), for which the odds ratio for ASD was 0.22 (95%CI: 0.07–0.62). 

The average results of questionnaires in the study group are presented in Table 2. In the analysis of the relationship between them and the genotypes of the studied polymorphisms within the study group, it was observed that holders of at least one allele of the mutant polymorphism rs10877969 obtained lower scores in terms of total ADOS-2 score (x_C_ = 14.90 (95%CI: 12.84–16.96) vs. x_T_ = 18.29 (95%CI: 16.68–19.90) *p* = 0.017) and social affect subscale (x_C_ = 12.38 (95%CI: 10.78–13.97) vs. x_T_ = 15.70 (95%CI: 14.27–17.13) *p* = 0.004). Carriers of the rs53576 mutant polymorphism allele had lower EQ scores (x_A_ = 15.2 (95%CI: 11.48–18.91) vs. x_G_ = 33 (95%CI: 20.29–45.70) *p* = 0.041), as well as significantly lower accuracy in RMiE (x_A_ = 60% (95%CI: 54–67%) vs. x_G_ = 81% (95%CI: 69–93%) *p* = 0.01). In addition, no other statistically significant differences were observed between the questionnaires depending on the genotype of the polymorphisms studied in the study group. Interestingly, in the case of the control group and rs10877969 polymorphism, significant differences were also observed in the results of the ADOS-2 and EQ tests: total result: x_CT_ = 3.93 (95%CI: 3.16–4.7) vs. x_CC_ = 1.33 (95%CI: 0.06–2.60) *p* = 0.002; social affect subscale: x_CT_ = 3.53 (95%CI: 2.69–4.36) vs. x_CC_ = 0.83 (95%CI: (−0.19) −1.86) *p* = 0.001; EQ: x_CT_ = 20 (95%CI: 16.15–23.84) vs. x_CC_ = 31 (95%CI: 24.98–37.01) *p* = 0.009.

After the completion of comparative analyses, a decision was made to deepen the analysis using multiple regression methods, focusing on genotype interaction models of selected polymorphisms with the result of the total ADOS-2 test and accuracy in the RMiE test. 

The first model was the relationship between the result of the total ADOS outcomes and subsequent polymorphisms in the study group. The regression coefficient was R2 = 0.3023 with *p* < 0.01576, and the standard estimation error Se = 3.77. Statistically significant predictors of ADOS-2 were the major variant of the polymorphism rs10877969 (β = −5.29; *p* < 0.05) and the major variant of the polymorphism rs7294536 (β = 4.68; *p* = 0.015). In the case of the social affect subscale (R2 = 0.342; *p* = 0.006; Se = 3.06), only the wild variant of the polymorphism rs10877969 (β = −4.40; *p* < 0.0001) was a significant predictor of outcome. Regression analysis for accuracy in the RMiE study showed no statistical significance in the study group. 

In the control group, statistical significance was also obtained for the result of the total ADOS-2 study (R2 = 0.5; *p* < 0.006; Se = 0.81) and the social affect subscale (R2 = 0.47; *p* = 0.01; Se = 1.45). In both models, the wild variant of the polymorphism rs10877969 was the statistically significant predictor, which obtained the parameter β = −1.51 (*p* < 0.05) for the total result and β = −2.69 (*p* < 0.05) for the social affect subscale, respectively.

## 4. Discussion

In terms of the analysis of the questionnaires used in the present study, attention is drawn primarily to the lack of a statistically significant difference in the percentage of correct responses within the RMiE survey between the study and control groups, as well as a slightly lower-than-expected, on the basis of previous studies, percentage of correct answers achieved in the entire population (64.7% vs. 69%11/72%15). This may indicate that disorders in the recognition of emotions based on the eyes, treated as a mechanism based on the theory of mind, may not be directly related to the clinical picture of ASD. Similar conclusions were drawn, for example, by Oakley et al. in a 2016 study [14], where in the comparison between a group of patients with ASD and neurotypical people, there were no statistically significant differences in the percentage of normal responses on the RMiE test. However, when analyzed independently of the ASD diagnosis, a significant difference in response rates was observed between patients with a high and a low outcome of alexithymia. Similar results were also obtained by i.e., Bird and Cook 2013 [15] or Ola and Gullon-Scott 2020 [16]. 

In terms of the results of the empathy quotient questionnaire in the presented analysis, a statistically significant difference was observed between the study and control groups. This was an expected difference, given the important role that the theory of mind plays in the mechanisms of cognitive empathy [17]. Furthermore, the analysis of the authors’ empathy quotient subscales showed the existence of average, statistically significant differences between groups of patients and controls. The magnitudes of the effect in the case of these differences were similar for AEQ and CEQ, which would indicate that, in the study group, both parameters were similarly disturbed compared to the neurotypical group. The presented data seem to be in line with previous research results summarized, e.g., by van der Zee and Derksen in 2020 [18], although they contradict some of the available literature—e.g., Smith et al. 2009 [19]—and seem to contradict the hypothesis of an imbalance between the elements of empathy as the basis of the clinical picture of ASD. On the other hand, the lack of a statistically significant difference between the groups in terms of the RMiE test, the lack of the expected difference between AEQ and CEQ, and the distribution of the result of the total ADOS-2 test in the study and control groups bring to mind the study of Camodeca et al. [20] and Miu et al. [21], where such phenomena were essential features of the so-called broad autism phenotype (broad autism phenotype (BAP)), i.e., a constellation of subclinical symptoms from the autism spectrum occurring in the population of neurotypical people. Available research indicates that this phenomenon may affect up to 25% of the population, and interestingly, contrary to the expectations of researchers, it did not show gender dependence [22]. This contributed to a deepening of research on the prevalence of “autism spectrum traits” in the general population and showed that their severity is consistent with the normal distribution in the general population [20]. This, in turn, naturally led to the conclusion that, in fact, the autism spectrum is a continuum in the general population, including both people completely devoid of this type of feature at one pole and with increasing severity of traits qualifying for the BAP group, then for a full diagnosis of ASD [23].

In the field of molecular studies among the examined children and adolescents with a diagnosis of ASD, a higher-than-expected frequency on the basis of the European/world population frequency of allele A rs53576 and allele C rs10877969 could be observed. For the group of children who did not achieve a diagnosis of ASD and did not exceed the cut-off threshold in the ADOS-2 study, a significantly higher incidence of the rs53576 A allele was also observed than would result from the reference frequency analyses. In the direct comparison between the study and control groups, a statistically significant difference in frequency was observed only in the case of the polymorphism rs10877969, which also translated into the results of logistic regression, where it was also the only polymorphism that significantly shaped the risk of ASD in the studied population. 

The AVPR1A gene, despite its close relationship to the OXTR gene and numerous contact points between the oxytocinergic and vasopresynergic systems in the CNS region, still remains relatively poorly studied [3]. In the present study, the polymorphism rs10877969 was the only one of the analyzed SNPs that significantly differed in allele distribution, not only from the expected distribution based on the European and world populations, both in the study and control groups, but also between the accepted study and control groups. In the study group, patients homozygous for the wild “T” allele had significantly higher scores in the ADOS-2 study than in the group of patients with at least one mutated allele. Similarly, in multiple regression analyses, the wild “T” allele of polymorphism rs10877969 was a statistically significant positive predictor of the “Social Affect” subscale result in the ADOS-2 study. In the control group, the mutant “C” allele rs10877969 was statistically significantly associated with lower comparative scores and lower scores on the “social affect” scale of the ADOS-2 study and a higher empathy quotient. In the analysis of works placed in the PubMed and APA PsycNet databases in the period from 2010–2021, 8 papers analyzing the relationships of various AVPR1A polymorphisms with social cognition and/or ASD disorders were identified, of which only 2 publications analyzed them together with the OXTR gene [24,25]. However, only two studies on the analyzed polymorphisms by Yang et al. 2010 [26] and Yang et al. 2017 [10] are available in the literature, which confirmed the data obtained in the present study, indicating the “T” allele as being associated with the risk of ASD and social cognitive impairment. Thus, both the data obtained in the course of the present study and the available, albeit poor, literature sources seem to indicate the existence of a relationship between the wild allele of the polymorphism rs10877969 and social cognition deficits in both patients and neurotypical individuals, as well as the existence of a relationship between this polymorphism and the risk of ASD. Interestingly, the genotype control group appeared to be “intermediate” between the reference population and the study group. 

The higher frequency of the A allele SNP rs53576 in the study group in comparison to the European and global populations indicated that it may be associated with the risk and clinical picture of ASD. In the available literature, this relationship also remains controversial. This was demonstrated, among others, in the literature review LoParo et al. [6] or in the analysis carried out by the author of the present study [3]. Only Wu et al. 2015 [27] has so far obtained results fully consistent with those presented in this study. In and of itself, the relationship between rs53576 and the ASD phenotype was also demonstrated by Liu et al. in 2010 [28], but in their work, it was at the limit of statistical significance (*p* = 0.053) and the wild allele, or “G”, was indicated as a risk factor. Other literature papers by Lerer et al. 2008 [29] and Harrison et al. 2015 [30], among others, did not show a relationship between the distribution of rs53576 alleles and the risk of ASD. Interestingly, despite the observed significant difference in the distribution of SNP rs53576 alleles in the ASD group compared to the European/world population, the present work, as most authors, showed no difference compared to the neurotypical control group. Consequently, the distribution of rs53576 alleles in the control group also deviated significantly from the expected values for the European and world populations. Similarly, in the logistic regression analysis, the value of the rs53576 genotype as a risk factor for ASD was also statistically insignificant. Assuming the hypothesis that SNP rs53576 is a risk factor for the development of ASD and impaired social cognition, the observed statistical relationships may indicate that, in the control group, despite the neurotypical character, there is an overrepresentation of people with discrete deficits in social cognition—which coincides with the results obtained from the questionnaire surveys. In the analysis of the relationship between elements of social cognition and the genotype rs53576, attention is drawn to the significant negative effect of the mutant allele (A) observed in this study on the results obtained by patients in the RMiE group. It is interesting that this type of relationship was observed only among patients diagnosed with ASD and not in the control group. This contradicts the reports of Weisman et al. [31], where the genotype AA and GA SNP rs53576 was associated with significantly lower RMiE test scores in the general population, but is consistent with the results of Lucht et al. 2012 [32], where the RMiE score among neurotypical subjects depended on the genotype rs2228485 in the OXTR gene, not on rs53576. This type of discrepancy may result from the fact that the result of the RMiE test may be affected by more than one polymorphism and not necessarily only in the OXTR gene. At the same time, in the research of Uzefovsky et al. [33], it was shown that the genotype rs53576 significantly affects the results of the RMiE test in the ASD population, which confirms the results obtained. Interestingly, the Uzefovsky study also showed that this polymorphism modulates brain activity in functional magnetic resonance imaging (functional magnetic resonance imaging (fMRI)) during the test. The changes in activity concerned primarily the right supramarginal gyrus and, in combination with the genotype of the polymorphisms studied (rs2254298, rs53576 and rs2268491), had a satisfactory ability to predict the diagnosis of ASD. 

In the SNP rs2254298, no significant differences in allele distribution were observed between the control, test, and expected distributions based on the European and world populations. This would be consistent with observations presented in several publications, including Lerer et al. 2008 [29] or Wermter et al. 2010 [34]. However, contrary data were presented, for example, in the studies by Liu et al. 2010 [28] and Francis et al. 2016 [24]. In a subsequent publication, Francis et al. 2016 [25], it was shown that haplotypes including SNP rs2254298 were significant. Finally, the results of the Friedlander et al. study from 2019 should also be recalled, where it was shown that each subsequent mutated allele in the main OXTR polymorphisms (including rs2254298) was responsible for the increasing severity of ASD features. Attention is therefore drawn to the high heterogeneity of the results available in the literature. In the context of the fact that the data obtained in the present study require the rejection of the hypothesis of a direct relationship between the genotype of the polymorphism rs2254298 and the pathogenesis of ASD, the results from the analysis of the literature raise the question of the real nature of the relationship between this SNP and the clinical picture of ASD and deficits in social cognition. Similarly, the polymorphism rs7294536 in the AVPR1a gene in the present study showed no significant differences between the test group and the control group and the reference population from the 1000Genomes database. Interestingly, in the analysis of multiple regression in the study group, in a model including both SNP rs10877969 and SNP rs7294536, it was observed that the mutated alleles of both polymorphisms significantly shaped the total result of the ADOS-2 test, but they did it in an opposite way—the mutated allele rs10877969 was associated with lower ADOS results and the mutant allele rs7294536 with higher. These results are also partially consistent with studies by Yang et al. 2017 [10] and Yang et al. 2010 [26], where the SNP genotype rs7294536 was significantly associated with ASD risk and showed numerous significant associations with impaired social communication and interpersonal relationship formation. In addition, for a promoter containing the mutant (“C”) allele rs7294536, Yang et al. [10] observed a significantly lower relative luciferase activity, indicating that this genotype may significantly affect gene expression for AVPR1A. 

Both results presented in this study and literature sources seem consistent in indicating that vasopressin receptor abnormalities are associated with the clinical picture and the risk of ASD. Furthermore, the obtained results are interesting in the context of the control group, which seems to occupy a somewhat intermediate place between the reference population and the study group. A potential explanation for this phenomenon, however, may be the inadequacy of the adopted dichotomous conceptualization of ASD, assuming the existence of an objectively separable group of “autistic” and “neurotypical” people. Assuming the opposite hypothesis, suggesting the existence of an autistic continuum in the population, we would obtain both an interesting explanation for the results obtained, as well as a solution to the problem of incompatibility of the conclusions drawn by different authors. In the case of genetic tests, this may be related to the mechanism by which, for example, the selected genes determine the occurrence of ASD. Considering the huge number of genes that show significant correlations with symptoms or spectrum risk [35,36,37,38], it can be assumed that the polymorphisms occurring in them determine certain areas of the clinical picture of ASD. However, none of them is sufficient to condition the occurrence of full-blown syndrome, and rather, as the number of these variants in a person’s genotype increases, the severity of autism spectrum traits would increase to a clinically relevant level. Of course, this relationship would not be linear. The increase in the number of “pro-autistic” alleles within genes conditioning a specific area of cognitive functions (e.g., social competences) would directly translate into the severity of these specific symptoms, rather than the whole picture of ASD. As a result, in the population, we would observe, firstly, significant variability in the clinical presentation of ASD between specific patients and, secondly, the occurrence of such symptoms and their relationship to these predefined “pro-autistic” variants in people who do not meet the formal criteria for the diagnosis of ASD. 

The main limitation of the present study is the small size of the included groups. However, taking into account the obtained statistical parameters, the existing distributions of the studied polymorphisms, and existing literature reports, the obtained results seem to reliably present a relationship between the “mass effect” of the increase in “proautistic” alleles of various polymorphisms in the population and the increase of quantitative and qualitative features from the autism spectrum. Another issue is the control for confounding factors. In this case, the main problem is the possible impact of polymorphisms not included in the analysis in other locations, which remain, for example, in an imbalance of couplings with the respondents and generate the risk of occurrence of apparent, statistically significant relationships with the analyzed parameters. 

## 5. Conclusions

1. The rs10877969 (T) polymorphism within the AVPR1a gene was the only one to show a statistically significant association with a higher risk of autism spectrum disorders and had an impact on clinical presentation in the ADOS-2 study, primarily in terms of the social affect subscale.
2. Polymorphisms in the OXTR gene showed no significant association with the ASD risk and severity of autistic traits in the ADOS-2 study.
3. In the group of people with ASD and neurotypical, the rs53572 (A) genotype in the OXTR gene significantly increased the severity of the clinical picture of social cognition disorders in the RMiE and EQ studies.

## Figures and Tables

**Table 1:** Distribution and frequency of alleles of selected polymorphisms in the test and control groups in relation to the reference frequency based on the 1000Genomes database [1,4].

		rs10877969 (AVPR1a)	rs53576 (OXTR)	rs7294536 (AVPR1a)	rs2254298 (OXTR)
Reference frequencies	Frequency of minor allele (MIF) in the world/European population	C = 0.2 /0.1312	A = 0.32646 /0.32577	C = 0.2969 /0.1312	A = 0.13978 /0.11288
	Major allele frequency (MAF) in the world/European population	T = 0.7033 /0.8688	G = 0.67354 /0.67423	T = 0.7031 /0.8688	G = 0.86022 /0.88712
Frequencies observed Study group	Frequency of the minor allele (MIF) in the study population (q)	C = 0.3462	A = 0.6282	C = 0.2179	A = 0.1026
	Major allele frequency (MAF) in the study population (P)	T = 0.6538	G = 0.3718	T = 0.7821	G = 0.8974
Frequencies observed Control group	Frequency of the minor allele (MIF) in the study population (q)	C = 0.6429	A = 0.6905	C = 0.1429	A = 0.0952
	Major allele frequency (MAF) in the study population (P)	T = 0.3571	G = 0.3095	T = 0.8571	G = 0.9048

**Table 2:** Comparison of the parameters of questionnaire survey results in the study and control groups; * Mann–Whitney U test: ES = effect size.

	Study Group		Control Group		
Test	Average	95%CI	Average	95%CI	ES; *p*-Value *
EQ	16.51	12.61–20.42	23.92	19.74–28.11	r = −0.38; *p* = 0.014
AEQ	5.62	4.25–7	8.28	6.55–10.01	r = −0.35; Q < 0.05
CEQ	10.88	8.24–13.53	15.42	12.6–18.22	r = −0.38; Q < 0.05
RMiE (%)	63.53	57.6–69.4	65.97	59.57–72.37	0.85

## Data Availability

The data presented in this study are available on request from the corresponding author. The data are not publicly available due to the ongoing data acquisition and analysis.
